# Deep learning-driven approach for cataract management: towards precise identification and predictive analytics

**DOI:** 10.3389/fcell.2025.1611216

**Published:** 2025-05-30

**Authors:** Shuaixin Lu, Lingling Ba, Jie Wang, Min Zhou, Peiyao Huang, Xiaohua Zhang, Simo Pan, Xinmiao Zhou, Kai Wen, Jing Sun

**Affiliations:** Tianjin Key Laboratory of Retinal Functions and Diseases, Tianjin Branch of National Clinical Research Center for Ocular Disease, Eye Institute and School of Optometry, Tianjin Medical University Eye Hospital, Tianjin, China

**Keywords:** artificial intelligence, deep learning, cataract, machine learning, convolutional neural network

## Abstract

Deep learning (DL) technology has shown significant potential in the whole process of cataract diagnosis and treatment through algorithms such as convolutional neural network (CNN). In terms of diagnosis, DL models based on fundus or slit-lamp images can automatically identify and grade cataract, and their diagnostic accuracy is close to or beyond the level of human experts. In the field of surgery, DL can analyze the operation video stage in real time, accurately track the instruments and optimize the operation process, and reduce the risk of intraoperative eye error through intelligent devices. DL could optimize the intraocular lens (IOL) power calculation, predict the risk of complications and long-term surgery requirements. However, insufficient data standardization, the “black box” characteristics of the model, and privacy ethics issues are still the bottlenecks in clinical application. In the future, it is necessary to improve the generalization ability of model through multimodal data fusion, federated learning and other technologies, and combine interpretable design (such as Grad-CAM) to promote the evolution of DL to a transparent medical decision-making tool, and finally realize the intelligence and universality of cataract management.

## 1 Introduction

With the swift advancement of artificial intelligence (AI) technology, Machine Learning (ML), particularly the subfield of DL, has offered new development prospects for ophthalmology. Its application scope has expanded from the initial diagnosis of fundus diseases (Oganov et al., 2023; Zhang et al., 2023a; Xu et al., 2024) (such as diabetic retinopathy, age-related macular degeneration, retinopathy of prematurity) to the detection of anterior segment diseases (Ting et al., 2021; Wu et al., 2022) (including glaucoma, cataract, iris and corneal diseases), and then to the management of ophthalmic surgeries and the evaluation of postoperative prognosis (Wang et al., 2022a). Cataract is the primary cause of blindness worldwide (Zhang et al., 2025). It is of great significance to incorporate DL into the entire treatment process of cataract.

DL has demonstrated a wide application prospect in cataract management. On the one hand, it is capable of reducing the global clinical burden and enhancing the efficiency and accuracy of clinical diagnosis and treatment by elevating the level of automation. On the other hand, it can transcend geographical limitations and align with the objective of the World Health Organization, thereby providing a broader, universal and precise medical service for the world, especially for low-income individuals in developing countries and remote areas, and reducing the rate of blindness caused by cataract. Currently, the application of DL in the domain of cataract mainly centers on recognition and prediction.

## 2 Identification

Image recognition algorithms based on DL typically use the CNN as the fundamental architecture. Due to its outstanding performance in image and video recognition, classification, and segmentation tasks, CNN has emerged as the most dominant DL algorithm framework in the domain of intelligent diagnosis of cataracts (Chen et al., 2022; Xie et al., 2023).

The core architecture of CNN consists of several key components (Lee et al., 2017):

(1) The Convolutional Layer, being the core component of the network, conducts sliding window operation on the input data via learnable filters (also referred to as convolutional kernels) to calculate the dot product between local regions and filters for achieving feature extraction. Each convolutional layer encompasses multiple independent filters, which are capable of detecting specific feature patterns in the input data, ranging from basic edge and corner features to complex texture patterns.
(2) The Activation Function typically follows the convolutional layer, and nonlinear activation functions such as Rectified Linear Unit are prevalently employed. These functions can not only enhance the expressive capacity of the network, enabling it to learn complex feature representations, but also endow the network with the ability to simulate more complex functional mappings by introducing nonlinear factors.
(3) The primary function of the Pooling Layer is to decrease the spatial dimension of the feature map, enhance the robustness of feature detection, and simultaneously reduce the number of parameters and computational complexity. Among them, max pooling is the most frequently utilized operation, which achieves the reduction of feature dimension by dividing the non-overlapping rectangular regions of the feature map and extracting the maximum value of each region.
(4) The Fully Connected Layer, typically located after multiple convolution and pooling layers, is characterized by the fact that each neuron establishes connections with all activation values of the previous layer and is capable of learning higher-level abstract feature representations.
(5) As an optimization component, the Normalization Layer has been widely employed in advanced network architectures such as Inception, which can effectively accelerate the training process and enhance the generalization ability of the model.

The outstanding performance of CNN in image processing tasks results from its distinctive mechanism. Firstly, the network is capable of automatically learning the spatial hierarchy of the image, gradually extracting from simple edge features to complex shape and object features. Secondly, due to the parameter sharing mechanism, the convolution kernel parameters are shared throughout the entire input data range. This not only significantly reduces the number of model parameters, effectively alleviates the overfitting problem, but also greatly enhances the computational efficiency. These characteristics make CNN the most representative DL architecture in computer vision.

### 2.1 Application progress of deep learning in the diagnosis of age-related cataract

Age-related cataract is one of the leading causes of visual impairment worldwide. According to the World Health Organization, although cataracts can be effectively treated through surgery, a considerable number of patients in low- and middle-income countries and rural areas fail to receive timely diagnosis and treatment due to the shortage of medical resources, inadequate screening equipment, and the scarcity of specialized ophthalmologists. Traditional cataract diagnosis mainly depends on professional ophthalmologists to assess the degree of lens opacity by using a slit lamp microscope. Although this approach is accurate, it demands high professional proficiency from doctors and is challenging to be popularized in areas with scarce resources. AI and DL algorithms offer new solutions for cataract diagnosis because of their efficient, economical, and scalable features (Zhao et al., 2024).

#### 2.1.1 Development of early DL diagnostic systems

Early studies mainly used traditional ML combined with DL to develop cataract diagnosis systems. In 2019, Hongyan Zhang et al. proposed a six-level cataract grading method, which categorized cataract into six grades: non-cataract, mild cataract, mild-to-moderate cataract, moderate cataract, relatively severe cataract, and severe cataract based on the degree of fundus image blurring caused by lens opacity (Zhang et al., 2019). A total of 1,352 fundus images taken by Topcon professional fundus camera were labeled by two ophthalmologists and verified by three experienced ophthalmologists. Based on this hierarchical approach, the research team developed a DL diagnostic system based on stacked multi-feature fusion. The ResNet18 network was utilized to extract high-level features, the gray level co-occurrence matrix was used to extract texture features, and two support vector machine (SVM) classifiers were employed as the basic learners, combined with the fully connected neural network for the final classification. The experimental results indicated that the average classification accuracy of the system reached 92.66%, and the highest was 93.33%, demonstrating the potential of DL technology in cataract diagnosis.

However, this classification method differs significantly from the Lens Opacities Classification System III (LOCS III) standard, which restricts its direct application in clinical practice. To solve this problem, Qiang Lu et al. developed an AI-assisted automatic cataract grading program based on the LOCS III standard and established a remote diagnosis platform (Lu et al., 2022). Utilizing advanced DL algorithms such as Faster Region-based Convolutional Neural Network (R-CNN) and ResNet, the program can precisely locate and analyze the region of interest (ROI) of the lens and performs well in nuclear and cortical cataract grading. In this study, 1,328 slit-lamp photographs from Eye & ENT Hospital of Fudan University from 2018 to 2020 and 355 slit-lamp photographs from Pujiang Eye Study from 2018 to 2019 were used as training, validation and test datasets. All images were labeled and verified by experienced ophthalmologists based on LOCSIII system. The results indicated that the absolute prediction error of the automatic nuclear cataract classification was 1.0 or less in 99.4% and 100% of the internal and external datasets, respectively, and the area under the curve (AUC) of referral ability was 0.983 and 0.977, respectively. Additionally, the AI program demonstrated good agreement with manual classification in nuclear and cortical cataract assessment, but there are still limitations in the classification of posterior subcapsular cataract (PSC).

#### 2.1.2 Data-driven optimization of DL algorithm

In order to improve the generalization ability and diagnostic accuracy of DL algorithm, researchers began to use larger data sets for model training. In 2022, Yih-Chung Tham et al. utilized over 25,000 fundus images from a population-based study to design and test a novel deep-learning algorithm for identifying visually significant cataracts (Tham et al., 2022). The AUC of the algorithm reached 96.6% in the internal test set and ranged from 91.6% to 96.5% in three external studies. Compared with ophthalmologists, the algorithm had a sensitivity of 93.3% and a specificity of 99.0%, which were superior to the performance of most ophthalmologists (sensitivity ranging from 51.7% to 96.6% and specificity ranging from 90.7% to 97.9%). This study indicates that DL algorithms, supported by large-scale datasets, are capable of attaining or even surpassing the diagnostic level of human experts.

Meanwhile, Tiarnan D.L. Keenan et al. developed a DL model, DeepLensNet, which was trained on the Age-Related Eye Disease Study dataset consisting of 18,999 slit-lamp photographs from 1,137 eyes of 576 patients (Keenan et al., 2022). DeepLensNet was designed to detect and quantify nuclear cataracts (NS), cortical cataracts (CLO), and PSC. The mean squared error (MSE) of DeepLensNet in the diagnosis of NS was 0.23, which was significantly better than that of ophthalmologists (MSE = 0.98) and medical students (MSE = 1.24). For CLO, DeepLensNet also outperformed human evaluators, but its accuracy was comparable to that of ophthalmologists in the diagnosis of PSC. This study indicates that the DL algorithm has significant advantages in the diagnosis of common cataract types, but further optimization is still necessary for the diagnosis of rare types.

#### 2.1.3 Challenges in the diagnosis of PSC

Although the DL algorithm demonstrates excellent performance in the diagnosis of NS and CLO, it still confronts a significant challenge in the diagnosis of PSC. In 2021, Elsa L.C. Mai et al. put forward a cataract shadow projection theory and attempted to utilize ultra-wide field photography and the DL algorithm to screen high-risk PSC (Mai et al., 2024). The research team developed a DL model based on ultra-wide field photography images to diagnose PSC by analyzing the shadow distribution of the lens. The study utilized 546 retrospective ultra-wide-field fundus images from Far Eastern Memorial Hospital (2018–2021), categorized into no shadow, blurry/cartwheel-like shadow, and central blotch shadow groups, and validated on a clinical dataset of 103 images. However, the experimental results indicated that the overall accuracy of the model was merely 80%, the sensitivity was 88.2%, and the specificity was 93.4%. This outcome suggests that the diagnosis of PSC remains a complex and challenging issue that might need to be addressed by combining multimodal imaging data with more advanced algorithms.

#### 2.1.4 Standardized image and model interpretability

In practical clinical applications, the generalization of the DL algorithm is restricted by insufficient standardized images and the “black box” problem of the model. In 2023, Eisuke Shimizu et al. put forward an innovative solution to increase the quantity of standardized optical images by using a video-recording slit-lamp device (Shimizu et al., 2023). The research team collected 206,574 video images from 1812 cataract eyes and employed the improved EfficientNet v2 model for training and testing. To enhance the interpretability of the model, Grad-CAM (gradient-weighted class activation mapping) was utilized to visualize the decision-making process of the model. Grad-CAM is a visualization technique designed to enhance the interpretability of deep learning models by highlighting the spatial regions of an input image that most significantly influence the model’s predictions. It achieves this by leveraging gradients of the target class score with respect to the feature maps from the final convolutional layer. Specifically, Grad-CAM computes gradient-based weights for each feature map channel, reflecting their importance to the model’s decision. These weights are then combined with the corresponding feature maps through a weighted summation, producing a coarse localization heatmap. The heatmap is subsequently upsampled to match the input image resolution and overlaid on the original image, offering an intuitive visualization of the regions the model prioritizes (Zhang and Ogasawara, 2023). Grad-CAM improves the model’s acceptability by highlighting the key regions in network predictions and assisting clinicians in understanding the diagnostic reasoning of AI. The experimental results indicate that the accuracy of the model in cataract diagnosis is significantly enhanced, and the application of Grad-CAM technology provides significant support for the promotion of AI in the medical field.

#### 2.1.5 Exploration of unsupervised learning

Traditional DL models typically depend on supervised or semi-supervised learning and demand a considerable amount of labeled data, which is costly in practical applications. In 2021, Yong Han et al. put forward an unsupervised anomaly detection model based on generative adversarial networks for the screening of eye diseases. The model was trained with 90,499 fundus images from various national and ethnic backgrounds and was capable of detecting multiple eye conditions, including cataracts (Han et al., 2021). The experimental results indicated that the AUC of the model in detecting cataract was 0.912, demonstrating the potential of unsupervised learning in the diagnosis of eye diseases. This approach not only lowers the cost of data labeling but also creates more possibilities for disease screening in low-resource settings.

#### 2.1.6 Innovation of anti-interference models

Image quality is a crucial factor influencing the performance of DL algorithms. In 2023, Xing Wu et al. developed an anti-interference AI model based on fundus images for the rapid and efficient diagnosis of cataracts (Wu et al., 2022). The model comprises two submodules: one for image quality recognition and the other for cataract classification. The quality recognition module can distinguish between normal and low-quality images and generate quality-related pseudo-labels for non-cataract images. The classification module classifies cataracts from superior-quality images based on CNN. The dataset which included 14,820 participants and 16,200 fundus images of cataract and noncataract was retrospectively derived from the Chinese PLA general hospital from September 2018 to May 2021. The experimental results indicate that the diagnostic accuracy of the model is significantly enhanced under low-quality images, providing a novel idea for addressing the issue of image quality.

Significant progress has been achieved in the application of AI and DL techniques to the diagnosis of age-related cataract. From early grading systems to optimization models based on large-scale datasets, and to innovations in unsupervised learning and anti-interference models, DL algorithms have gradually tackled the problems of data standardization, model interpretation, and image quality. In the future, with the further advancement of technologies such as multimodal data fusion, transfer learning, and federated learning, AI is anticipated to become an important tool for cataract diagnosis, especially in areas with scarce resources, and make greater contributions to global eye health.

### 2.2 Application of deep learning in the diagnosis of pediatric cataract

Pediatric cataract is one of the main causes of blindness among infants and young children globally (Solebo et al., 2018; Rodriguez et al., 2023). Its early diagnosis and effective treatment are crucial for preventing vision impairment. In recent years, DL has demonstrated significant potential in the diagnosis and management of pediatric cataract, offering innovative solutions to this field.

#### 2.2.1 A DL diagnostic system for pediatric cataract based on slit-lamp images

Xiyang Liu et al. initiated a computer vision framework based on slit lamp images and CNN for the (Liu et al., 2017) automatic localization and diagnosis of pediatric cataract. This framework enables the automatic analysis of slit lamp images by identifying the ROI of the lens and integrating the CNN algorithm. Specifically, the lens’ ROI was automatically located through Canny edge detection and Hough transform techniques, and then trimmed and adjusted to a fixed-size image for the establishment of a pediatric cataract dataset. The experimental results indicated that the average accuracy, sensitivity, and specificity of the proposed method in the classification task reached 97.07%, 97.28%, and 96.83% respectively. The proposed method also performed well in the three-degree classification task (including area, density, and location), with the average accuracy being 89.02%, 86.63%, and 90.75% respectively. This study provides an essential technical foundation for the automated diagnosis of pediatric cataract.

#### 2.2.2 Improvement of generalization ability of multi-center data

Despite the remarkable results of single-center studies, the versatility of DL diagnostic systems is limited due to the complex noise and heterogeneity of slit lamp images from multi-centers. To address this issue, Jiewei Jiang et al. developed two lens segmentation strategies based on DL Faster R-CNN and Hough transform to improve the generalization ability of infant cataract detection (Jiang et al., 2021). The data of this study were obtained from the Zhongshan Ophthalmic Center of Sun Yat-sen University. A total of 886 slit-lamp images were collected, of which 476 were normal images and 410 were images of children with different degrees of cataract. The experimental outcomes reveal that the average intersection ratio of Faster R-CNN in normal and abnormal lens segmentation tasks is 0.9419 and 0.9107 respectively, with an average accuracy of 95%. Compared with Hough transform, the accuracy, specificity and sensitivity of Faster R-CNN in the classification of opaque regions were increased by 5.31%, 8.09% and 3.29% respectively. Additionally, the processing time of a single image by Faster R-CNN is merely 0.25 s, which is significantly superior to that of Hough transform (34.46 s). Through Grad-CAM and t-Distributed Stochastic Neighbor Embedding techniques, the research team also accomplished the visualization of the lesion area and discrimination of high-level features, further strengthening the interpretability of the model.

DL technology has shown a broad application prospect in the diagnosis and management of pediatric cataract. From the automatic diagnosis system based on slit lamp images to the generalization ability improvement of multi-center data, DL algorithm has gradually solved the key problems in the diagnosis and treatment of pediatric cataract. In the future, with the further advancement of technology and multidisciplinary collaboration, AI is anticipated to play a more significant role in the early diagnosis, precise treatment and long-term management of pediatric cataract, and make a crucial contribution to the cause of global children’s eye health.

### 2.3 Integrated application of cataract diagnosis system with portable devices, telemedicine and personalized management subsequent to cataract surgery

With the rapid development of DL technology, its application in the field of ophthalmology has expanded from traditional diagnosis to surgical assistance and telemedicine. Especially in the diagnosis, management, and identification of the surgical stage of cataract, the combination of DL technology with portable devices and telemedicine platforms has brought revolutionary alterations to ophthalmic medicine.

#### 2.3.1 Portable devices facilitate the development of telemedicine in remote areas

Traditional fundus photographs and slit-lamp examinations rely on specialized equipment and ophthalmic technicians, limiting their uptake in low-resource Settings. However, technological advancements in smartphone cameras present novel prospects for eye examinations. Shenming Hu et al. developed a smartphone-based portable slit lamp intended to offer an early cataract screening tool in remote and deprived areas (Hu et al., 2020). The device simplifies the band design of the traditional slit lamp by integrating a miniature lens with a mobile phone camera to fulfill the portability requirements. This cost-effective and user-friendly device provides significant support to primary care.

#### 2.3.2 Personalized management and complication prediction combined with telemedicine platform

Erping Long et al. developed an AI agent named CC-Guardian for personalized management and complication prediction in patients with congenital cataract (Long et al., 2020). CC-Guardian encompasses three functional modules: a prediction module, a scheduling module, and a telemedicine module. The prediction module analyzed the baseline information (such as gender, age at surgery, and eye), lesion (such as area, density, and location), comorbidities (such as strabismus, microphthalmia), and surgical procedures (such as lens extraction and posterior continuous circular capsulotomy) via the Bayesian algorithm to identify high-risk patients who might have complications. The scheduling module formulates personalized follow-up plans based on the predicted outcomes, for instance, adding additional follow-up time points for high-risk patients. The telemedicine module utilized the deep residual network (ResNet-101) to analyze the postoperative images and offer intervention decision support. Clinical records of 594 patients with congenital cataract were included. A total of 4,881 postoperative follow-up images were collected. The performance of the model was further evaluated by internal validation (142 patients) and multi-resource validation (79 patients). The developed telemedicine platform enables real-time interaction between patients and specialized medical centers through cloud and smartphone applications, which can help doctors dynamically adjust follow-up plans and provide intervention decisions.

The research team explored neural networks and random forest algorithms within the prediction module; however, ultimately, it was discovered that the naive Bayes algorithm boasted lower computational complexity while maintaining a high level of accuracy, and was capable of achieving swifter decisions and greater stability. The application of CC-Guardian significantly enhances the quality of follow-up management for patients with congenital cataract, mitigates the social and economic burden of patients and their families, and offers a novel strategy for chronic disease management.

### 2.4 Application of deep learning for cataract surgery recognition

DL models can precisely identify different stages of cataract surgery by learning from cataract surgery videos, providing an essential tool for surgical training and process optimization. Groundbreaking research in this field includes.

#### 2.4.1 Real-time surgical stage segmentation and classification

Gwenole Quellec et al. developed a real-time system based on video content retrieval, which is capable of segmenting cataract surgery videos into idle phases and action phases (Quellec et al., 2014). The system employs conditional random fields for classifying action phases and predicting the next action phase. The study used data from 23 retinal surgeries (69 videos), 100 cataract surgeries (900 videos) and 1707 Hollywood film clips annotated with 12 human movements from the University Hospital of Brest, France, to identify eye surgery tasks and general behaviors through real-time video analysis. In the “Injection” and “Coat” recognition tasks in retinal surgery, the AUC of the proposed method reached 0.923 and 0.995, respectively, which was significantly better than that of the benchmark method. It performed well in the Phacoemulsification and Epinucleus removal tasks of cataract surgery (AUC = 0.923 and 0.969, respectively). The proposed method achieved 26.0 FPS, 24.3 FPS and 28.9 FPS in the three datasets, respectively, which was close to real-time (25 FPS). However, it has the drawback of insufficient recognition of complex tasks, such as “viscoelastic agent injection,” which involves subtle fluid movement, and the AUC is only 0.561. Moreover, the training is time-consuming, taking an average of 16 h for each task (based on a 12-core processor), which limits the practical application. Overall, the proposed system performed well in real-time segmentation and classification tasks, providing an efficient tool for automated analysis of surgical procedures.

#### 2.4.2 Performance comparison of multiple algorithms

Felix Yu et al. conducted a comparison of the performance of 5 ML algorithms in the classification of cataract surgery video stages, encompassing SVM, recurrent neural network (RNN), CNN, and the CNN-RNN combination model (Yu et al., 2019). The study used 100 videos of cataract surgeries (29 performed by senior surgeons and 71 by trained surgeons) from the Johns Hopkins Wilmore Eye Institute between July 2011 and December 2017, with 10 surgical stages and 14 device usage manually annotated as benchmark data. The findings indicated that the weight-free accuracy of these algorithms ranged from 0.915 to 0.959, and the area under the AUC ranged from 0.712 to 0.773. Among them, the AUC value of the CNN-RNN model on image data (0.752) was significantly higher than that of using CNN alone (0.712). Despite the high specificity among all algorithms (ranging from 0.877 to 0.999), the sensitivity range was broad (varying from 0.005 to 0.974), suggesting that DL techniques possess significant advantages in the recognition of cataract surgery stages.

#### 2.4.3 Automatic annotation system for surgical tools

Hassan Al Hajj et al. devised a DL-based automatic annotation system for cataract surgical tools in a competitive format (Al Hajj et al., 2019). The study employed 50 cataract phacoemulsification surgery video datasets, and a total of 14 teams submitted 27 protocols. Ultimately, the average recognition AUC value of the best-performing algorithm reached 0.9971, demonstrating the high efficacy of the DL algorithm in surgical tool recognition. This technique not only facilitates the postoperative self-evaluation of the surgeon but also optimizes the surgical workflow and offers the possibility of real-time intraoperative feedback.

DL models are capable of identifying various stages of cataract surgery through the learning of cataract surgery videos, thereby offering a crucial tool for surgical training and process optimization.

### 2.5 A breakthrough in the clinical application of cataract surgery recognition

The application of DL technology in cataract surgery has moved from theoretical research to clinical practice, including surgical skills assessment, surgical safety improvement and intelligent operating room management.

#### 2.5.1 Objective evaluation of capsulorhexis technique

The DL model developed by Tae Soo Kim et al. allows for an objective assessment of capsulorrphy techniques through the analysis of tool tip position, tool tip velocity, and optical flow field in surgical videos (Kim et al., 2019). The video recordings of 99 cataract surgery procedures were used as the data source. The technical skills in the videos were labeled by an expert surgeon according to the International Council of Ophthalmology Surgical Competence Evaluation criteria (ICO-OSCAR:phaco), and the instrument tip trajectory was marked by a crowdsourcing tool. The study found that the deep learning model using tool tip velocity (TV) had the best performance in the evaluation of cataract surgery capsulorruption technology, with an accuracy of 84.8% (AUC 86.3%), while the model based on optical flow field (FF) only had an accuracy of 63.4% (AUC 80.3%). However, combining multiple data features, such as FF + TV, did not significantly improve the performance. The model is capable of scoring and predicting the surgeon’s capsulorhexis technique, thereby providing a quantitative index for surgical skills training.

#### 2.5.2 Smart speakers reduce the risk of eye error

Tae Keun Yoo et al. have designed a DL-based smart speaker for confirming surgical sites during surgical intermission (Yoo et al., 2020). In this study, the CNN model was combined with the public Speech Commands dataset (containing more than 65,000 short audio messages) and 16 self-recorded target words (such as “left”, “right”, and “cataract”). The model was trained by data augmentation (including sound amplitude adjustment, time translation, and mixed operating room noise). The Adam optimizer (initial learning rate 0.0002, decay every 20 epochs) and dropout technique (probability 0.2) were used to prevent overfitting. Finally, the model was deployed on the LattePanda development board with commercial speakers, and passed 200 speech pauses during simulated surgery. The identification accuracy (93.5%) and the identification ability of key surgical information (100%) were verified. Through voice-controlled interaction, this device avoids the risk of infection caused by operators touching non-sterile areas and significantly reduces the possibility of eye error. The experimental data indicate that the accuracy of the device is nearly 100%, providing a guarantee for the safety of surgery.

#### 2.5.3 Preoperative safety management system

The preoperative safety management system for cataract surgery developed by Gaku Kiuchi et al. integrates facial recognition, surgical side confirmation, and verification of IOL parameters (Kiuchi et al., 2022). In a clinical trial involving 171 patients, the certification rate of facial recognition was 92.0% on the first attempt and rose to 96.3% after four attempts. The certification rate of the first attempt for the surgical side was 82.5%, and climbed to 98.2% after seven attempts. For the verification of IOL parameters, the initial and final certification rates were 67.4% and 88.9% respectively. Although the certification rate of IOL parameter validation did not reach 100%, the system was still regarded as a potent safety guarantee tool.

#### 2.5.4 Real-time surgical guidance and intelligent operating room management

Rogerio Garcia Nespolo et al. successfully integrated a surgical microscope with an AI platform via deep neural networks, being capable of real-time pupil tracking, identifying the surgical stage, and activating surgical guidance tools (Garcia et al., 2022). In this study, regional convolutional neural network (Faster R-CNN) combined with ResNet-50 architecture was used to process surgical video frames in real time. Through pupil tracking and surgical stage identification, computer vision tools (such as optical flow tracking, k-means clustering segmentation, and contrast enhancement) were linked to provide real-time visual feedback and warning for surgeons. The developed artificial intelligence platform based on the regional convolutional neural network (Faster R-CNN) attained a high level of accuracy in pupil tracking (with a Dice score of 90.23%) and surgical stage identification (with a maximum AUROC of 0.997) during cataract surgery, featuring a processing speed of 97 frames per second. 72% of the participating surgeons concurred on its usefulness in complex surgery, and all the participants affirmed that real-time feedback contributed to enhancing surgical safety. The immediacy of this method offers a new potential for the management of future intelligent operating rooms and can provide significant support for the surgical training of junior doctors.

The combination of DL technology with portable devices and telemedicine platforms provides a new solution for cataract management. From portable slit-lamp to surgical stage recognition, to intraoperative real-time feedback and training support, DL technology is gradually changing the practice patterns of eye care. In the future, with the further maturity of technology and the deepening of multidisciplinary collaboration, DL is expected to promote the popularization and precision development of ophthalmic medicine on a global scale.

## 3 Prediction

### 3.1 Optimization of predicted IOL power calculation

The accuracy of IOL power calculation directly influences the refractive effect after cataract surgery (Wang and Koch, 2021). The development of the IOL formula has undergone a transformation from theoretical optical models to those based on data and algorithms. The introduction of AI technology has promoted the calculation accuracy. Wiktor Stopyra et al., in their 2024 review, evaluated multiple AI-based formulas for IOL power calculation, including FullMonte, Ladas Super formula AI, PEARL-DGS, Kane, Karmona, Hoffer QST, and Nallasamy formula, etc (Stopyra et al., 2024). This study analyzed 25 peer-reviewed articles using the mean absolute error (MAE) and the percentage of patients within ±0.5 D as evaluation indicators and found that Kane’s formula performed best in terms of accuracy and became the most reliable AI-based formula for IOL power calculation. This progress has provided patients with additional accurate predictions of postoperative refractive outcomes and significantly improved surgical satisfaction.

### 3.2 Prediction of axial IOL position after cataract surgery

The accurate prediction of the axial position of IOL is the key to determine the refractive effect (Kane and Chang, 2021; Keshav and Henderson, 2021; Schallhorn et al., 2021). Achim Langenbucher et al. used DL algorithms and multiple linear regression models to predict ALP based on preoperative biometric data (Langenbucher et al., 2022). The study utilized the SVM and Gaussian process regression algorithm to measure 1,345 biometric data measured by IOLMaster 700. The results indicated that the root mean square prediction error (RMSE) of the Gaussian process regression algorithm was 0.2731 mm, and the MAE was 0.1948 mm. It outperformed the traditional multiple linear regression model (RMSE = 0.3379 mm, MAE = 0.2415 mm). This study demonstrates that DL algorithms possess significant advantages in handling nonlinear relationships and offer a novel approach for the accurate prediction of the postoperative refractive effect.

### 3.3 Prediction of the likelihood of requiring cataract surgery in the future

Wei Wang et al. gathered multidimensional demographic, socioeconomic, medical history, and lifestyle data through a questionnaire administered to 207,573 participants aged 45 or above with no history of cataract surgery (Wang et al., 2022b). Random forest, gradient-boosted ML, DL (multilayer feedforward neural network), and traditional logistic regression models were employed to predict the 10-year risk of cataract surgery. The findings indicated that the gradient boosting algorithm and random forest model demonstrated the best prediction performance (AUC≈0.78–0.79), which was significantly superior to the traditional logistic regression model. Age, subjective vision, and health insurance were core predictors. This study reveals that AI algorithms can be utilized in public health resource allocation planning and high-risk population screening, providing a novel strategy for the prevention and control of cataract.

### 3.4 Visual acuity prediction after cataract surgery in patients with high myopia

Patients with high myopia are often accompanied by complex fundus lesions, thereby making the prediction of postoperative vision more challenging (Wei et al., 2021). Ling Wei et al. developed a prediction system based on a DL model to predict the postoperative best corrected visual acuity by using preoperative macular optical coherence tomography (OCT) images (Wei et al., 2021). The study was based on preoperative OCT image data of 1,415 cataract patients with high myopia from Eye & ENT Hospital of Fudan University and 161 cataract patients with high myopia from Shanghai Peace Eye Hospital. Five pre-trained CNN models, namely, ResNet-18/34/50/101 and Inception-v3, were employed in this study, in combination with ensemble learning methods. The results indicated that the MAE and RMSE of the ensemble learning model in the validation set were 0.1566 logMAR and 0.2433 logMAR respectively. The advantage of this model lies in the fact that it only requires a single-mode OCT input and has strong clinical applicability. The prediction sensitivity for the good visual acuity group is over 80%, and it can provide decision support for more than 60% of patients. This technique offers an important reference for preoperative communication and surgical planning in patients with high myopia.

### 3.5 Risk prediction of posterior capsule opacification after phacoemulsification

Posterior capsular opacity is the most common complication after cataract surgery, and over 20% of patients need Yttrium Aluminum Garnet laser capsulotomy. Seyed-Farzad Mohammadi et al. gathered 10 influencing factors (such as age, gender, diabetes, type of IOL, etc.) of 352 eyes after cataract surgery and constructed a decision tree, back propagation artificial neural network, and logistic regression model (Mohammadi et al., 2012). The results indicated that the back propagation artificial neural network model had the highest prediction accuracy (87%), and the area under the ROC curve was 0.71, which was significantly superior to the decision tree model (accuracy 50%) and the logistic regression model (accuracy 88%). Studies have demonstrated that DL models have significant advantages in handling complex interactions and multi-factor predictions, providing an efficient tool for Posterior capsular opacity risk prediction.

The application of DL in cataract surgery, ranging from the optimization of IOL power calculation to the prediction of postoperative complication risks, has demonstrated its significant potential in enhancing surgical accuracy and patient satisfaction. With the further maturation of technology and the deepening of multidisciplinary collaboration, it is anticipated that AI will promote the development of personalization, precision, and intelligence of cataract surgery on a global scale, bringing more benefits to both patients and doctors.

## 4 Challenges and discussion of DL in cataract management

The research on the combination of AI and cataract diagnosis has been going on for more than 10 years. Rapid and accurate diagnosis is no longer a problem (Chen et al., 2025), and the prediction of postoperative effects and complications has become a new research hotspot. Internet Plus Healthcare has emerged, while there are a number of issues that can be discussed that hinder the application to the Clinical practice.

### 4.1 Data security and privacy protection

As a discipline deeply associated with imaging, the medical data of cataract patients encompass a considerable amount of highly sensitive content, such as patient identity information, biological characteristics, and disease history. The training of DL models depends on a large quantity of such data (Abràmoff et al., 2022). The “productization” of trained AI models in the clinical setting is extremely intricate, and data might need to be shared among multiple institutions, perhaps even across countries, during the process of facilitating telemedicine and smart health (Chaet et al., 2017). With the expansion of the transmission scale, the risk of patient privacy leakage is directly magnified. If the patient’s medical data is not fully desensitized (for instance, not concealing the name or examination number), the attacker can restore specific individual information from the training set through model reverse engineering or adversarial sample attack, or even combine it with external databases (such as medical insurance records) to achieve secondary identification. Cybersecurity measures will become increasingly crucial to address the risks of improper utilization of data sets, inaccurate or inappropriate disclosure, and limitations of de-identification techniques. The current healthcare environment lacks the impetus for data sharing, and the ownership and usage rights of patients’ desensitized medical data require further legal and ethical deliberations (Yu et al., 2018).

It has been suggested to establish anonymous “benchmark datasets” with known diagnoses, which are regularly updated and “calibrated” using local data from the implementing facility, similar to the way clinical laboratories maintain local reference standards for blood-based biomarkers. Clearly, these maintenance measures entail extensive data sharing and considerable manpower. Local calibration is of significance because some algorithms might have local or culture-specific parameters that may not be applicable to different populations. Future research should prioritize transparent reporting of demographic metadata and imaging protocols to facilitate robust meta-analyses.

Vulnerabilities in data storage and transmission are equally fatal. When hospitals collaborate with third-party technology companies, if the original data stored centrally does not employ homonymous encryption or secure multi-party computing, once the cloud server is invaded, patient privacy might be exposed on a large scale. If real-time data transmission in remote diagnosis and treatment lacks quantum encryption or blockchain traceability technology, man-in-the-middle attacks can intercept unencrypted pathology reports or real-time monitoring data. Additionally, although distributed technologies such as federated learning can achieve “data non-discharge,” malicious nodes may still restore the patient’s original data from the shared gradient updates through gradient inversion (Teo et al., 2025). A more insidious risk lies in the security threat after the model is deployed. If an attacker implants a backdoor trigger (such as a specific pixel combination) during the training stage, the AI diagnostic system can misjudge the nature of the disease when faced with imaging pictures containing the trigger, directly endangering the patient’s life (Nazir and Kaleem, 2023).

### 4.2 Interpretability of the model

The application of the DL model in cataract diagnosis and treatment encounters significant interpretability challenges. The DL model has black-box characteristics because of its complex interactive multi-level nonlinear structure and autonomous feature learning mechanism (Oganov et al., 2023). In the medical context of cataract, models extract features through tens to hundreds of layers of neural networks. For instance, hidden features such as tissue texture and lesion contour are gradually abstracted from the pixel-level data of eye OCT images (Kashani et al., 2023).

Taking the opacity of the eye lens as a typical case, the model might generate prediction results based on the nonlinear combination of millions of parameters, but it fails to clearly explain why an abnormal increase in density is regarded as a cataract. This ambiguity of the decision path directly gives rise to a crisis of confidence in clinical practice. It is also impossible to rule out the possibility that the model is misled by irrelevant noise (Kihara et al., 2022).

In order to address this dilemma, researchers are exploring two approaches, namely, “*post hoc* interpretation” and “self-interpretation model”. The former employs Grad-CAM and other technologies to visualize the model’s focus areas in imaging images, enabling clinicians to compare whether the model’s recognition focus aligns with the basis of clinical diagnosis (Zhang and Ogasawara, 2023). In the task of lesion classification, the model not only outputs the diagnostic results but also presents the similarity comparison with the lesion features of typical cases. Nevertheless, the above two methods still have their own limitations, and the accuracy and stability require improvement.

Therefore, the development of AI medicine is moving towards a new paradigm of “interpretable embedding,” which demands the introduction of medical prior knowledge constraints from the early stage of model design. This includes the construction of interpretable modules in the prediction model or the cross-modal alignment of pathology report text and image features. In the future, AI medical systems are required to possess both diagnostic capabilities and teaching abilities. They should not only assist in decision-making but also clarify their reasoning logic to doctors by visualizing decision trees and feature contribution heat maps, ultimately achieving the paradigm shift from “black box” to “transparent diagnosis and treatment partners” (He et al., 2019).

### 4.3 Ethical and moral issues

The rapid advancement of AI has enhanced the efficiency of cataract screening; however, it has also sparked multi-dimensional ethical disputes in clinical practice. The crux of these disputes lies in the clash between technology application and medical humanistic value. When algorithmic decision-making is involved in the diagnosis and treatment of human’s most sensitive sensory function, the underlying reconfiguration of power and transfer of medical responsibility are challenging the traditional ethical framework (He et al., 2019).

A more insidious risk lies in the implicit transfer of clinical decision-making power. The accuracy of current cataract diagnostic models is higher than that of junior residents and junior doctors. When it is put into practical application, doctors may blindly adopt algorithm suggestions and give wrong diagnosis (Kermany et al., 2018). However, the ambiguity regarding responsibility for medical accidents has not been clearly defined in the current legal system, and how hospitals, algorithm developers, and cloud server providers should divide their rights and responsibilities has been exposed, highlighting the lag of legal norms (Oganov et al., 2023).

The ethical dilemma brought about by the application of the DL model is essentially the profound impact of technical rationality on humanistic medical care. Cataract patients frequently exhibit cognitive anxiety regarding body datalization during the selection and planning of IOL before surgery, which reflects the fundamental contradiction between the requirements of individualized diagnosis and treatment and the simplification of algorithmic thinking in the process of modern medical technology. This contradiction is not only manifested at the level of technical cognition but also has a profound influence on the trust foundation of the doctor-patient relationship.

The “third path” for the construction of AI ethics in cataract requires the establishment of a multi-dimensional framework. Regarding technical interpretability, a traceability model with anatomical relevance should be developed. Concerning clinical application standards, it is essential to establish a quality control system including uncertainty assessment. In the reconstruction of the doctor-patient relationship, an innovative model combining algorithm transparency and patient empowerment should be explored. Although these practical explorations did not completely solve the ethical dilemma, they provided an important practical path for the ethical development of cataract AI models.

### 4.4 Future research directions

Future technological optimization will focus on enhancing the personalized prediction capabilities and generalization performance of models, which can be achieved by expanding the database through collecting population information from multiple regions and ethnic groups and fusing multimodal data (Oke, 2022). The construction of cross-regional multi-center benchmark datasets should follow strict standardized protocols. Regulations similar to the “Cataract Image Acquisition Specifications” should be formulated, and equipment parameters (such as slit lamp light source intensity and shooting angle), data formats (DICOM standard), and annotation criteria (LOCS III classification) should be unified. The dataset should cover diverse races, ages, and disease stages, and emerging subtypes (such as diabetic cataract) should be regularly included. To protect patient privacy, a dynamic calibration mechanism can be implemented through a federated learning framework, where each institution regularly uploads local data statistical features (such as the mean distribution of lesions), and the central model adjusts the weights accordingly to avoid performance degradation due to population differences (Moshawrab et al., 2023). Multimodal data fusion can be achieved by integrating ophthalmic images, genomic data, environmental factors, and patient electronic health records to construct a multi-dimensional feature space. However, this method needs to address the issue of data heterogeneity, such as developing cross-modal alignment algorithms to unify the representation forms of different data sources (Zhang et al., 2023b).

Clinical translation can focus on intelligent surgical robots and real-time decision support systems. Intelligent surgical robots are the key direction for the next technological breakthrough. Current surgical robot systems are mostly auxiliary operation systems that rely on pre-set programs and lack real-time decision-making capabilities during surgery (Moglia et al., 2021). In the future, by embedding reinforcement learning algorithms, robots can autonomously handle unexpected situations during surgery (such as posterior capsule rupture). Real-time decision support systems during surgery should deeply integrate image stream analysis and physiological signal monitoring. For example, based on real-time surgical video streams under the microscope, the system can identify the hardness of the lens nucleus and recommend the best phacoemulsification mode; or through continuous monitoring of intraocular pressure sensors, the system can warn of the risk of suprachoroidal hemorrhage. The application of this system also needs to be combined with edge computing, moving data processing from the cloud to local devices (such as microscopes embedded with GPUs) to reduce the impact of network latency on real-time performance.

## 5 Conclusion

The extensive application of DL technology in the diagnosis and treatment of cataract is propelling ophthalmic medicine into the era of precision and intelligence. Through algorithms such as the CNN, DL not only enables the automatic classification and diagnosis of cataract, significantly enhancing the efficiency and accuracy of screening, but also demonstrates excellent potential in surgical stage identification, tool tracking, and postoperative complication prediction. Particularly in low-resource areas, the intelligent platform combining portable devices and telemedicine offers a universal solution for the prevention and treatment of cataract worldwide. Nevertheless, the technology still confronts challenges like data standardization, model interpretability, privacy security, and ethical controversy.

Future research should focus on technological optimizations such as multimodal data fusion, federated learning, and transfer learning to enhance the generalization ability and clinical adaptability of the model. Meanwhile, an interdisciplinary collaboration mechanism should be established, the data privacy protection framework should be improved, and doctor-patient trust should be enhanced through the design of “interpretable embedding”. Only by striking a balance between technological innovation and ethical governance can DL be truly integrated into clinical practice, providing more efficient, safe, and humanized diagnosis and treatment services for cataract patients and contributing to the long-term development of global eye health.
